# Biocontrol of Biofilm Formation: Jamming of Sessile-Associated Rhizobial Communication by Rhodococcal Quorum-Quenching

**DOI:** 10.3390/ijms22158241

**Published:** 2021-07-31

**Authors:** Yvann Bourigault, Sophie Rodrigues, Alexandre Crépin, Andrea Chane, Laure Taupin, Mathilde Bouteiller, Charly Dupont, Annabelle Merieau, Yoan Konto-Ghiorghi, Amine M. Boukerb, Marie Turner, Céline Hamon, Alain Dufour, Corinne Barbey, Xavier Latour

**Affiliations:** 1 Laboratory of Microbiology Signals and Microenvironment (LMSM EA 4312), University of Rouen Normandy, F-27000 Evreux, France; yvann.bourigault@univ-rouen.fr (Y.B.); chane.andrea@gmail.com (A.C.); mathilde.bouteiller7@univ-rouen.fr (M.B.); charly.dupont7@univ-rouen.fr (C.D.); annabelle.merieau@univ-rouen.fr (A.M.); yoan.konto-ghiorghi@univ-rouen.fr (Y.K.-G.); amine.boukerb@univ-rouen.fr (A.M.B.); corinne.barbey@univ-rouen.fr (C.B.); 2 Research Federations NORVEGE Fed4277 & NORSEVE, Normandy University, F-76821 Mont-Saint-Aignan, France; 3 Laboratoire de Biotechnologie et Chimie Marines, LBCM IUEM, EA 3884, Université de Bretagne-Sud, F-56100 Lorient, France; sophie.rodrigues@univ-ubs.fr (S.R.); laure.taupin@univ-ubs.fr (L.T.); alain.dufour@univ-ubs.fr (A.D.); 4 Laboratoire Ecologie et Biologie des Interactions, UMR CNRS 7267, F-86073 Poitiers, France; alexandre.crepin@univ-poitiers.fr; 5 Vegenov, F-29250 Saint-Pol-de-Léon, France; turner@vegenov.com (M.T.); hamon@vegenov.com (C.H.); 6 Biocontrol Consortium, F-75007 Paris, France

**Keywords:** communication, biofilm, lifestyle switch, *Rhizobium* (*Agrobacterium*) *rhizogenes*, hairy root, quorum-sensing, *N*-acyl homoserine lactones, *Rhodococcus erythropolis*, quorum-quenching, biological control

## Abstract

Biofilms are complex structures formed by a community of microbes adhering to a surface and/or to each other through the secretion of an adhesive and protective matrix. The establishment of these structures requires a coordination of action between microorganisms through powerful communication systems such as quorum-sensing. Therefore, auxiliary bacteria capable of interfering with these means of communication could be used to prevent biofilm formation and development. The phytopathogen *Rhizobium rhizogenes*, which causes hairy root disease and forms large biofilms in hydroponic crops, and the biocontrol agent *Rhodococcus erythropolis* R138 were used for this study. Changes in biofilm biovolume and structure, as well as interactions between rhizobia and rhodococci, were monitored by confocal laser scanning microscopy with appropriate fluorescent biosensors. We obtained direct visual evidence of an exchange of signals between rhizobia and the jamming of this communication by *Rhodococcus* within the biofilm. Signaling molecules were characterized as long chain (C_14_) *N*-acyl-homoserine lactones. The role of the Qsd quorum-quenching pathway in biofilm alteration was confirmed with an *R. erythropolis* mutant unable to produce the QsdA lactonase, and by expression of the *qsdA* gene in a heterologous host, *Escherichia coli*. Finally, *Rhizobium* biofilm formation was similarly inhibited by a purified extract of QsdA enzyme.

## 1. Introduction

Bacteria are rarely encountered as single dispersed organisms in the environment. They generally live in communities and may colonize the surfaces of minerals and living tissues by forming biofilms [1,2,3]. This process involves the formation of aggregates with distinct sessile cells, followed by cell division to form small clusters, microcolonies, and larger aggregates. During the maturation step, the microbial community synthesizes a hydrated matrix of polysaccharides, proteins, and nucleic acids, in which bacterial cells are embedded. Finally, only the underside of the biofilm remains in direct contact with the substrate, the rest of the biofilm forming a multilayer heterogeneous microbial mat [1,2,4,5,6]. Switching to this lifestyle provides the microbes with more favorable environmental conditions, favoring their survival in an otherwise hostile environment (i.e., dehydration, oligotrophy, and the presence of antibiotics and predators), leading to a sustainable colonization of the coveted niche, in addition to avoiding their washing out (e.g., in a flowing stream or an infected gut) [1,3,7]. 

The biofilm phenotype may be seen as a form of collective behavior, in which all the members of the community work together to ensure the persistence of the group within the environment [8,9,10,11]. For example, bacteria express different phenotypes according to their location within the structure of the biofilm. The bacteria at the periphery can be in an active metabolic state, enabling them to shield the ‘core bacteria’ at the center of the biofilm, including the dormant persister cells [9]. This social behavior requires finely tuned coordination between members of the biofilm. The switching of bacteria between motile behavior and a sessile lifestyle, including biofilm formation, maturation, and/or dispersion, therefore requires a plethora of molecular tools, including environmental sensors (two-component systems), quorum-sensing (QS) networks, and intracellular messengers such as 3′,5′-cyclic diguanylic acid (cyclic di-GMP) [12,13,14,15,16]. *N*-acyl-l-homoserine lactone (AHL)-based QS networks are strongly suspected to function as a master regulator of biofilm development in many Gram-negative bacteria [13,17,18,19]. In this communication system, each individual constitutively produces diffusible AHL signals, the environmental concentration of which is, therefore, directly linked to the density of the emitting population, but also its location, as in a host wound, which favors signal accumulation. Bacterial populations capable of detecting AHLs are, therefore, informed about the number of cells (quorum) and the degree of diffusion from the microenvironment, between bacterial sites within and outside the biofilm, for example. QS also enables the bacteria to synchronize the expression of genes involved in biofilm synthesis, the production of ‘public goods’ and other cooperative traits with benefits available to all the cells of the population at an individual cost to the responding cell [8,11,19,20]. 

As QS is essential for biofilm installation and development, disrupting the flow of signals between biofilm members by a quorum-quenching (QQ) mechanism appears to be a promising method of control [21,22,23,24,25,26,27]. This possible application of QQ first emerged in the clinical context [21,22,23,24] and in the field of anti-biofouling [25,26,27,28]. It has since been more explicitly proposed as a biofilm control method in more recent reviews [7,29,30]. QQ encompasses all the processes involved in the disturbance of QS, mediated by QS inhibitors, and by enzymes capable of inactivating QS signals, known as QQ enzymes [31,32]. Some environmental microorganisms can synthesize acylases, oxidoreductases, and/or lactonases, which then modify the various parts of AHLs [27,33,34,35,36]. In contexts in which lifestyle switches and biofilm formation are determinant traits for plant disease, the development of an anti-biofilm strategy essentially amounts to the development of an anti-virulence strategy, mediated by beneficial microorganisms acting as biocontrol agents [27,37,38,39]. 

Recent studies have shown that AHL-based QS disruption by both QQ inhibitors and enzymes can effectively reduce biofilm formation [21,40,41], but our knowledge remains limited by the paucity of direct visual evidence of the jamming (i.e., disturbance) of QS communication and its effects on biofilm formation. We aimed to bridge this gap by studying the biofilm formed by the *α*-Proteobacterium *Rhizobium rhizogenes* (formerly *Agrobacterium rhizogenes*). This bacterium is a soilborne pathogen that causes hairy root disease in susceptible dicotyledonous plants [42,43,44,45]. It induces anarchic growth of the root system after wounding and infection. *R. rhizogenes* introduces its root growth-inducing transfer DNA (Ri T-DNA) into the host plant genome, bypassing the plant defense system, and then modulates the endogenous levels of two hormones, auxin and abscisic acid, leading to the initiation and proliferation of hairy roots [45,46,47]. Hydroponically grown tomato (*Solanum lycopersicum* L.) and cucumber (*Cucumis sativus*) plants are greatly affected by this disease, which results in large economic losses [43,44,48]. In these cropping conditions, pathogenic strains of *R. rhizogenes* hijack the metabolism of the plant, directing it towards root production at the expense of aerial apical growth and fruit production. They also produce massive biofilms in the greenhouse irrigation system, which may obstruct the supply circuits with saline solutions (biofouling), thereby affecting crop yields [42,43,44,48]. AHL-based-QS systems are common in rhizobia [49,50,51,52]. We therefore hypothesized that AHL molecules control biofilm formation in *R. rhizogenes*. We validated this hypothesis by using the Gram-positive bacterium *Rhodococcus erythropolis* R138 to counteract biofilm formation by *R. rhizogenes* [53,54]. This biocontrol agent effectively degrades diverse AHLs, reducing levels of potato (*Solanum tuberosum*) blackleg and soft rot in hydroponic and field conditions [54,55]. In this strain, full QQ activity requires expression of the QS signal degradation (Qsd) pathway, leading to the production of the QsdA and QsdC intracellular enzymes, involved in the lactone ring and acyl chain catabolism of AHLs, respectively [53]. 

Confocal laser scanning microscopy (CLSM) can be used to observe the structure of biofilms and to quantify their biovolumes and thicknesses. It can also be used to monitor the QS and QQ phenomena occurring within the biofilm through the use of biosensors carrying promoter-probe vectors fused to reporter genes encoding fluorescent proteins. We used a dual-color strain of *R. erythropolis*, R138, for simultaneous observation of the disruption of AHL communication and its impact on the biofilm. This strain constitutively produces the mCherry fluorophore as a cell tag, as well as a reporter fusion based on the green fluorescent protein (GFP) gene, making it possible to detect AHL signaling molecules and their degradation simultaneously [38,53]. Using these biosensors, we provide the first images of QS and QQ molecular interactions within biofilm. Finally, we demonstrate the close connection between biofilm development and QS communication by highlighting the capacity of the lactonase QsdA both to silence AHL communication and to prevent biofilm formation. 

## 2. Results

### 2.1. Characterization of the AHL Signaling Molecules Produced by R. rhizogenes

The type strain of *R. rhizogenes* species (*R. rhizogenes* CFBP 5520^T^, hereafter named *R. rhizogenes* 5520^T^) was used as a model in our study. The AHL signals produced by this strain were identified by analyses of stationary phase cultures in lysogeny broth (LB; syn. Luria–Bertani broth). AHLs were extracted from culture supernatants with ethyl acetate and analyzed by high-performance liquid chromatography coupled with mass spectrometry (HPLC–MS).

**Figure 1:**
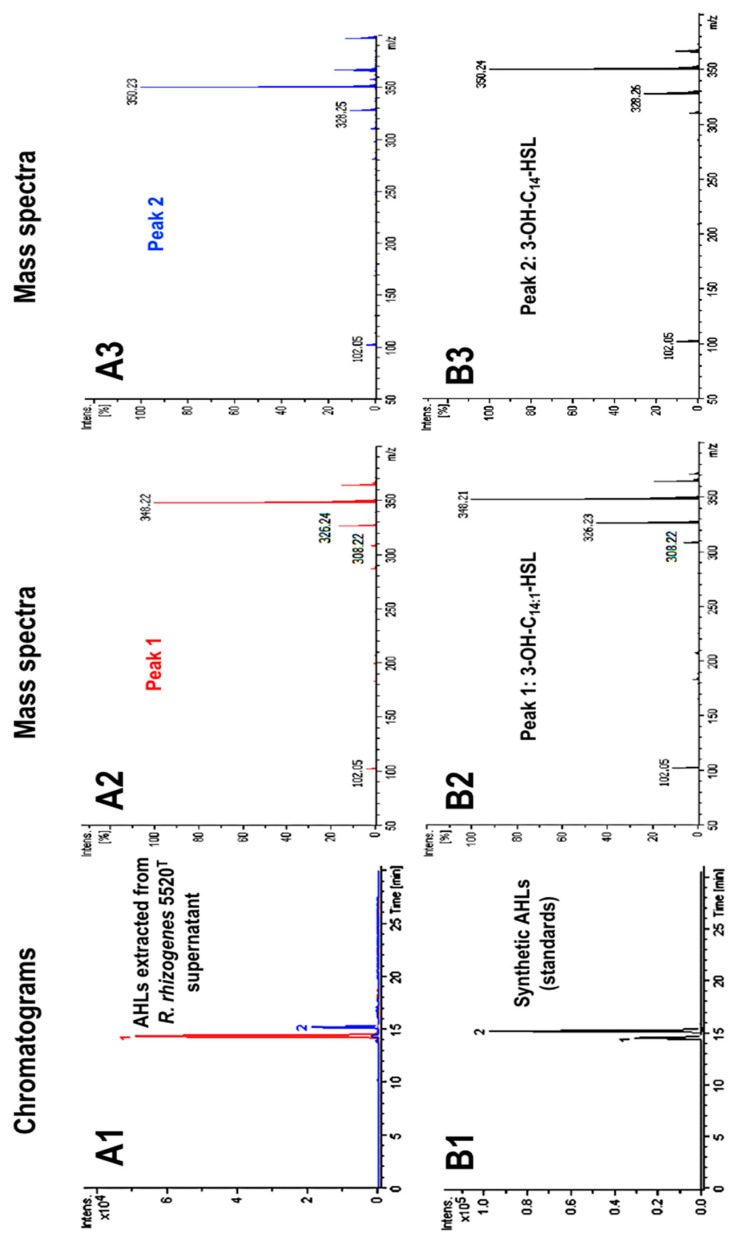
HPLC–MS analyses of AHL signaling molecules in *R. rhizogenes* culture supernatants. The chromatogram of an AHL extract from strain 5520^T^ (**A1**) and associated mass spectra of compounds from chromatogram peaks 1 (**A2**) and 2 (**A3**) were compared to the chromatogram of a mixture of two synthetic AHLs, 3-OH-C_14:1_-HSL and 3-OH-C_14_-HSL (**B1**), and the corresponding mass spectra of compounds from chromatogram peaks 3 (**B2**) and 4 (**B3**).

In mass spectrometry analyses, a fragmentation product at *m*/*z* 102.05 is characteristic of a lactone ring, and, thus, constitutes the signature of an AHL molecule. The extract contained two compounds yielding such fragmentation products. These two compounds were eluted at retention times of 14.3 and 15.2 min on the HPLC chromatogram (Figure 1A1). The mass spectrum for compound 1 revealed molecular [M+H]^+^ and [M+Na]^+^ ions at *m*/*z* 326.24 and 348.22, respectively, and fragmentation products at *m*/*z* 102.05 and 308.22 (Figure 1A2). This pattern is consistent with an AHL, as shown by the presence of a lactone ring (*m*/*z* 102.05), with a total molecular weight (*M*_W_) of 325 Da. The latter excludes the fact that the AHLs detected harbored a short or medium aliphatic chain. On the basis of this *M*_W_, compound 1 may be either *N*-(3-oxotetradecanoyl)-l-homoserine lactone (3-oxo-C_14_-HSL) or *N*-(3-hydroxy-tetradecenoyl)-l-homoserine lactone (3-OH-C_14:1_-HSL) with one unsaturated bond in the acyl chain, both of which have the same *M*_W_. The [M+H]^+^ fragment at *m*/*z* 308.22 indicated that this *R. rhizogenes* AHL was probably 3-OH-C_14:1_-HSL, because hydroxylated AHLs characteristically lose water during MS analyses. Compound 2 (Figure 1A1) yielded a mass spectrum with molecular [M+H]^+^ and [M+Na]^+^ ions at *m*/*z* 328.26 and 350.24, respectively, and the lactone ring fragmentation product at *m*/*z* 102.05 (Figure 1A3). This compound, with a *M*_W_ of 327 Da, could be 3-OH-C_14_-HSL.

For the above results showing that *R. rhizogenes* AHLs contain a long (C_14_) acyl chain, we used the three synthetic AHLs with C_14_ acyl chains to confirm our identification conclusions and identify which of these C_14_ AHLs were indeed produced by *R. rhizogenes*. The synthetic AHLs were 3-oxo-C_14_-HSL, *N*-(3-hydroxy-7-cis-tetradecenoyl)-l-homoserine lactone (3-OH-C_14:1_-HSL), and *N*-(3-hydroxy-tetradecanoyl)-l-homoserine lactone (3-OH-C_14_-HSL). We analyzed them in the same HPLC–MS conditions as above for the strain 5520^T^ extracts. Synthetic 3-OH-C_14:1_-HSL and 3-OH-C_14_-HSL yielded peaks at the same retention times, 14.3 and 15.2 min, respectively, as the two natural AHLs (Figure 1B1). Moreover, the molecular [M+H]^+^ and [M+Na]^+^ ions and fragmentation products resulting from these two synthetic AHLs (Figure 1B2,B3) were consistent with those of the *R. rhizogenes* AHLs (Figure 1A). By contrast, synthetic 3-oxo-C_14_-HSL had a retention time of 15.9 min, and its mass spectrum was clearly different from those shown in Figure 1B2,B3 (see Figure S1). In conclusion, *R. rhizogenes* 5520^T^ produces two AHLs, identified in these analyses as 3-OH-C_14:1_-HSL and 3-OH-C_14_-HSL. Using standards at different concentrations, we showed that 3-OH-C_14:1_-HSL was about 10 times more abundant than 3-OH-C_14_-HSL (Figure 1A1).

### 2.2. Characterization of the Structure of the Biofilm Generated by R. rhizogenes and Inhibition by the Biocontrol Agent R. erythropolis R138

**Figure 2:**
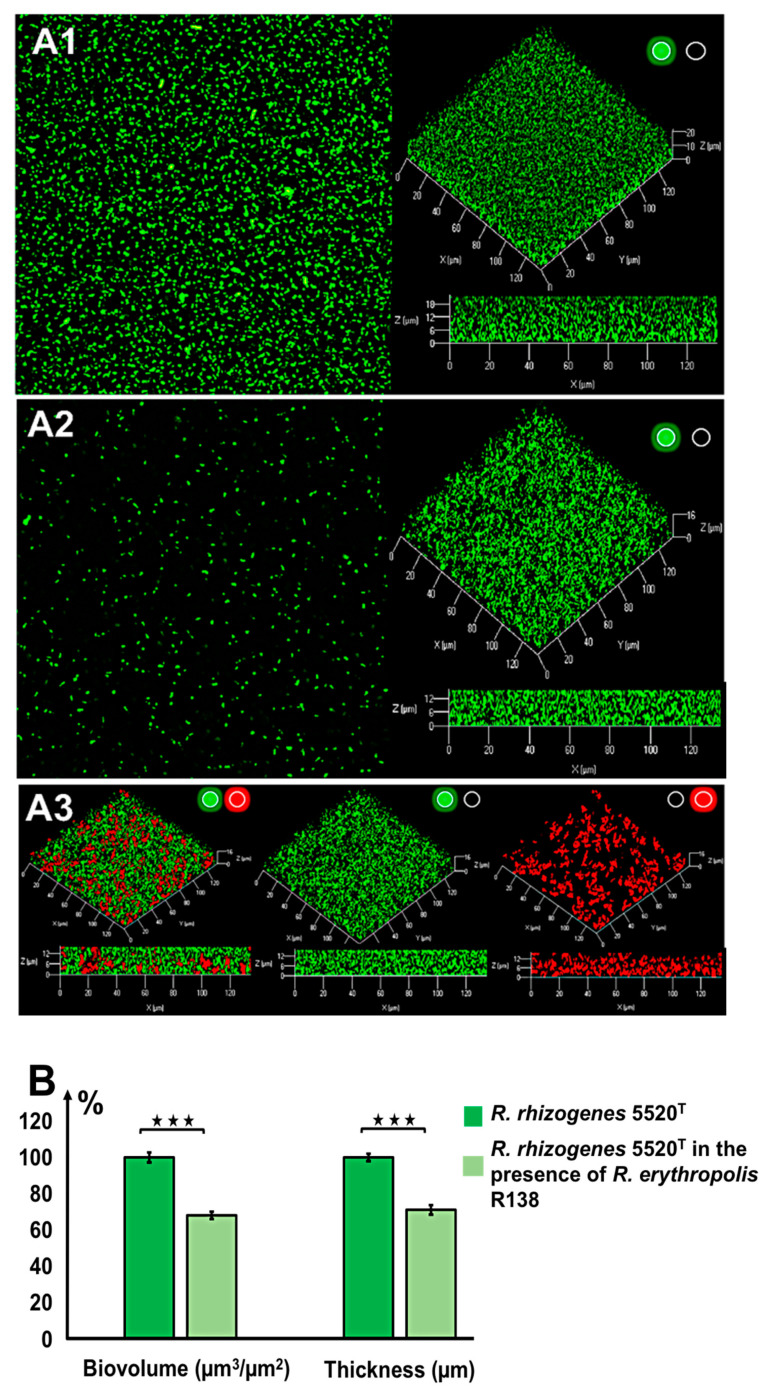
Inhibition of rhizobial biofilm formation by the biocontrol agent *Rhodococcus erythropolis* R138 (ratio 1:1). Biofilms were developed during incubation at 25 °C for 48 h in LB under static conditions. Confocal laser scanning microscopy (CLSM) analysis of the *R. rhizogenes* 5520^T^ biofilm (**A1**) or dual-species biofilm formed by *R. rhizogenes* 5520^T^ (green fluorescence) and *R. erythropolis* R138 (red fluorescence) (**A2**,**A3**) was achieved at an inoculation ratio of 1:1. *R. rhizogenes* and rhodococcal bacteria were tagged with green fluorescent protein (GFP) and mCherry via the pHC60-*gfp* and pEPR1-*mCherry* vectors, respectively. Top view, 3D shadow representation, and side view of the biofilm produced by *R. rhizogenes* alone (**A1**), and by a mixed culture of *R. rhizogenes* and *R. erythropolis* (**A2**) analyzed in the green channel. Location of rhodococcal cells was revealed by 3D shadow representation and side view of the biofilm produced by the mixed culture analyzed in the red plus green, green, or red channels (**A3**) *(activation of the green or red channel is indicated by a light spot of the corresponding color).* COMSTAT analyses of *R. rhizogenes* green fluorescence in single- and dual-species biofilms. *R. rhizogenes* biofilm biomass and thickness values were normalized (set to 100%) as a reference for a comparison with mixed biofilm conditions (**B**). The data shown are the means of three measurements from three independent experiments. Significant differences (Mann–Whitney test; *p*-value < 0.01) in biovolume and thickness are indicated by asterisks (★★★ *p* < 0.001).

For microscopy analyses of *R. rhizogenes* biofilm formation, the GFP-encoding pHC60-*gfp* plasmid was introduced into the *R. rhizogenes* 5520^T^ strain (for details of all tagged strains, biosensors, and plasmids, see Table S1). CLSM analysis revealed that this pathogen formed a thick, compact, homogeneous layer on glass slides (Figure 2A). The biovolume of the biofilm, reflecting the amount of biomass present, was measured, together with biofilm thickness, in a COMSTAT analysis [56]. After culture in static conditions for 48 h at 25 °C, the biofilm of strain 5520^T^ was 24.2 ± 0.8 µm thick and had a biovolume of 23.54 ± 0.10 μm^3^/μm^2^.

No direct antagonism between the phytopathogen *R. rhizogenes* 5520^T^ and the biocontrol agent *R. erythropolis* R138 was observed during in vitro assays in solid medium (Figure S2). Given this apparent compatibility, we assessed the formation of a dual-species biofilm by mixing a culture of *R. rhizogenes* 5520^T^ pHC60-*gfp* with culture of *R. erythropolis* R138, in a 1:1 ratio (on the basis of bacterial counts), as described in the Materials and Methods section. For the simultaneous visualization of these two strains by CLSM, the pEPR1-*mCherry* vector was introduced into *R. erythropolis* R138, resulting in the constitutive tagging of this strain with red fluorescence (Table S1).

Following activation of the green channel only, to detect the green fluorescence of *R. rhizogenes* in the mixed biofilm, we found the biofilm to have a more thinner structure than the standard *R. rhizogenes* biofilm formed in the absence of *R. erythropolis* (Figure 2A). Moreover, the roughness coefficient of the mixed biofilm, established using Comstat2 software, showed a significant positive change in comparison to the control biofilm, 0.019 ± 0.004 for dual species biofilm vs. 0.006 ± 0.001 for the rhizobial biofilm, indicating an increase in the biofilm heterogeneity (Figure S3). This alteration of biofilm structure is also illustrated in detail with pixel density analysis in Figure S4. Indeed, pixel mean values related to GFP fluorescence, representing rhizobial cell density, were lower for each stack of the dual species biofilm than those recorded for the single species biofilm (Figure S4). For example, at the bottom of the biofilm core (stack n°4), the pixel mean value ranged from 57.13 ± 3.25 to 30.37 ± 3.83, corresponding to a decrease of 47% (Figure S4). Analysis of the 3D representation and side view of mixed biofilm revealed the presence of *R. erythropolis* aggregates harboring the expected red fluorescence. These aggregates were scattered inside the biofilm developed by *R. rhizogenes* and appeared to be interposed between *R. rhizogenes* layers (Figure 2A). The presence of the R138 strain in the mixed biofilm led to a decrease in the biovolume of the *R. rhizogenes* strain from 22.57 ± 0.47 to 15.39 ± 0.56 µm^3^/µm^2^, corresponding to a 31% decrease in biovolume. Mean biofilm thickness also decreased, from 21.85 ± 0.37 to 15.53 ± 0.42 µm, corresponding to a 29% decrease (Figure 2B). The use of an inoculum with the 5520^T^ vs. R138 ratio shifted in favor of strain R138 (1:10) for biofilm formation amplified these changes by a factor 1.87 for biovolume and 1.84 for thickness (Figure S5). These findings indicate that the biocontrol agent R138 clearly alters both the structure and dimensions of the *R. rhizogenes* biofilm.

### 2.3. Production of AHL Signaling Molecules by R. rhizogenes within the Biofilm

The formation of a dual species biofilm was assessed by mixing a culture of the *R. rhizogenes* 5520^T^ strain with the *P. atrosepticum* 6276-EI AHL-biosensor strain. For microscopic visualization, both strains were labeled in red by staining nucleic acid with syto61. The *P. atrosepticum* 6276-EI AHL-biosensor has no AHL synthase of its own (*luxI* mutant) and carries a lux-based reporter system consisting of a transcriptional fusion of the *luxI* promoter and the *gfp_ASV_* gene encoding an unstable GFP derivative. This system is under the control of the *luxR* (regulator) gene. Thus, in the presence of AHLs, the quorum sensor LuxR binds to the *luxI* promoter region and activates the transcription of the *gfp_ASV_* gene. This induces bright-green fluorescence in the biosensor strain as soon as a minimum AHL concentration of 10 nM is reached [38,57].

**Figure 3:**
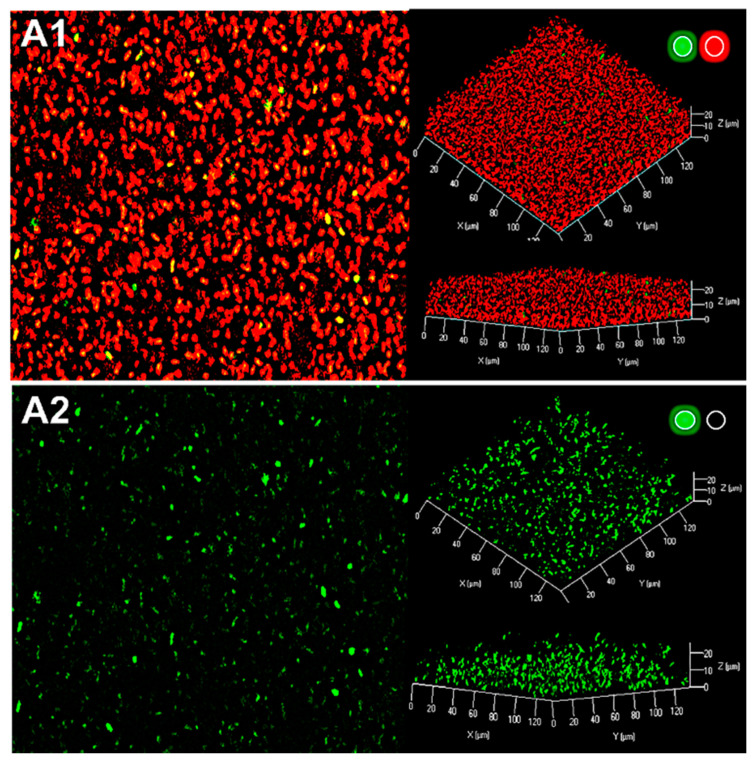
Production of rhizobial AHL signaling molecules within the biofilm. CLSM analysis of the dual-species biofilm formed by *R. rhizogenes* 5520^T^ and the *P. atrosepticum* 6267-EI AHL biosensor strain (1:1 ratio). Bacteria were labeled with the nucleic acid stain Syto 61 (bright red fluorescence), whereas the *P. atrosepticum* 6276-EI AHL-biosensor was also tagged by GFP, due to the *gfp* expression governed by the AHL inducible promoter. Top view, 3D shadow representation and side view of the biofilm produced by a mixed culture of *R. rhizogenes* and *P. atrosepticum* (**A1**,**A2**). We analyzed the structure of the biofilm (bright red fluorescence) due to the activation of the green plus red channels for (**A1**), and used the green channel only for (**A2**), for visualization of the presence of AHL (bright green fluorescence) in the biofilm through the expression of the *gfp* reporter gene under control of the AHL-inducible promoter (*activation of the green or red channel is indicated by a light spot of the corresponding color*).

Analyses of the 3D representation and side view of the mixed biofilm showed that *P. atrosepticum* was present as yellow or green spots dispersed throughout the biofilm developed by *R. rhizogenes* (Figure 3). In our assay conditions, *Pectobacterium* was present as single coccobacillus cells or, more rarely, as small microcolonies, mostly in the core of the biofilm, with the single cells preferentially located towards the top or the base of the biofilm. We found that *P. atrosepticum* 6276-EI was a poor biofilm former and colonizer, and therefore had only a very limited influence *R. rhizogenes* biofilm formation, consistent with its expected role as a biosensor. The *P. atrosepticum* cells emitted a green fluorescence, revealing the presence of AHLs throughout most of the biofilm. As the *P. atrosepticum* 6276-EI sensor cannot itself produce AHLs [38,58], the signals detected can only have been synthesized through the QS communication of rhizobia within the biofilm.

### 2.4. Quenching of R. rhizogenes AHL-Based Communication within the Biofilm by the Biocontrol Agent R. erythropolis R138

The biosensor strain *R. erythropolis* R138 carrying the pEPR1-*qsdR*-P*qsd::gfp-mCherry*, a vector mimicking the system regulating the *qsd* operon [53], was used to monitor QQ activity in a mixed biofilm. In addition to the *mCherry* cassette under the control of a constitutive promoter, this vector contains a *qsdR-*P*qsd::gfp* transcriptional fusion composed of the *qsd* promoter upstream from the *gfp* ORF and the QsdR repressor-encoding gene (Table S1). In these conditions, the absence of *gfp*-expressing bacteria (i.e., of red fluorescent cells) is due to the repression of *qsd* promoter activity by QsdR. The presence in the environment of various AHLs leads to binding of the AHL-homoserine lactone ring to the transcriptional repressor QsdR, preventing the physical binding of QsdR to the P*qsd* promoter, and resulting in expression of the *gfp* gene. The combination of GFP and mCherry fluorophores tags the bacteria with yellow to amber fluorescence, according to the intensity of QQ activity [38,53,59]. A previous CLSM study of this biosensor cultured with different concentrations of AHL-inducers or analogs found that the threshold concentration for the induction of QQ activity was about 1 μM [38,53].

**Figure 4:**
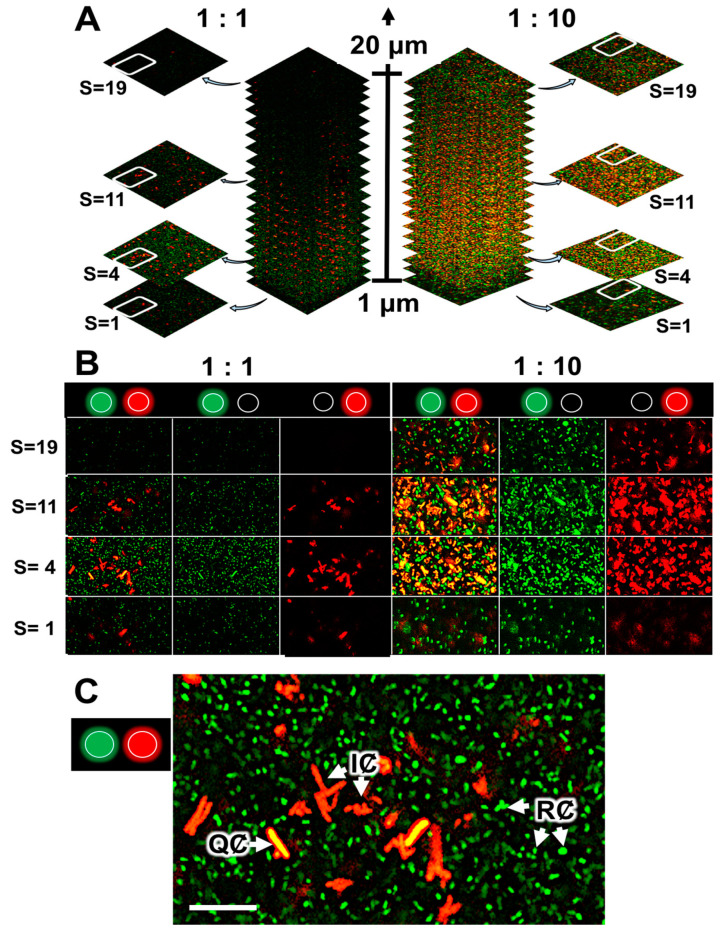
CLSM analysis of quorum-quenching activity in a dual-species biofilm of *R. rhizogenes* and *R. erythropolis*. *R. rhizogenes* (green fluorescence) was tagged with GFP via the pHC60-*gfp*. *R. erythropolis* was transformed with the pEPR1-*qsdR*-P*qsd::gfp-mCherry* vector containing a transcriptional fusion between the promoter of the AHL lactonase-encoding gene *qsdA* and the *gfp* gene for the monitoring of its quorum-quenching activity. (**A**) A total of 19 confocal image stacks acquired with 1 µm slices from the bottom to the top of the mixed biofilms. For each mixed biofilm (with an *R. rhizogenes*/*R. erythropolis* ratio of 1:1 at left or 1:10 at right), four image stacks taken at 1 (S = 1), 4 (S = 4), 11 (S = 11), and 19 (S = 19) µm from the bottom of the biofilm were selected for the assessment of rhodococcal quorum-quenching activity. (**B**) Details delineated by the white circled area of four sample image stacks, previously selected among images in (**A**), to highlight the rhodococcal quorum-quenching activity at the different observation channels. (**C**) Close-up of the selected image stack S = 4 with an *R. rhizogenes*/*R. erythropolis* ratio of 1:1. Legend: RȻ, rhizobial cells (green fluorescence), QȻ, quenching cells sensing and degrading *N*-acyl-homoserine lactone (AHL) signals (green plus red, i.e., yellow, fluorescence); IȻ, inactive cell not sensing or degrading AHL signals (red fluorescence) (*activation of the green or red channel is indicated by a light spot of the corresponding color*). Scale bar represents 5 µm.

We investigated the formation of mixed biofilms with 1:1 and 1:10 ratios (in terms of bacterial counts) of *R. rhizogenes* (labeled in green fluorescence) and *R. erythropolis* (constitutively marked in red fluorescence and inducibly labeled in yellow in the presence of AHLs) (Figure 4A). Distribution of the rhizobial and rhodococcal cell patterns was evaluated through pixel mean values analysis, related to GFP and mCherry fluorescence, respectively. It revealed that even if rhizobial and rhodococcal cells were evenly distributed throughout the biofilm, only few rhodococcal cells were found at the base of the biofilm, implicated in adhesion to the surface (Figure S6). Indeed, an analysis of 2D images focusing on a specific area of the biofilm (Figure 4A, stacks 4 to 11, corresponding to the core of the biofilm) showed that the core contained a mixture of red and yellow *R. erythropolis* cells. In this context, yellow fluorescence was more readily observed in a mixed biofilm established with a 1:10 ratio of the two species, consistent with the larger number of *R. erythropolis* GFP-emitters (Figure 4A). ‘False-positive’ yellow cells, due to an overlap of green fluorescent rhizobia and red fluorescent rhodococci, were excluded from the analyses by the use of several controls. First, it was possible to distinguish between the green fluorescence associated with the GFP (tag) and GFP_UV_ (QQ activity) fluorophores produced by the rhizobial and rhodococcal cells, respectively, by breaking the overall image down according to the signals obtained in the various channels (Figure 4B). Second, it is possible to check that cells are not superimposed on each other by comparing the different stacked planes of the biofilm with the z-stacks CLSM tool (Figure 4A). Finally, at high magnification, it remains possible to distinguish clearly between clustered cells, rod-shaped rhizobia and the adjacent filamentous rhodococci (Figure 4C). It was, therefore, possible to monitor the ability of certain *R. erythropolis* cells to perceive and degrade AHL molecules by assessing yellow fluorescence, with inactive cells remaining red. The findings obtained confirmed that AHLs were produced within the biofilm (see Section 2.3) and induced promoter activity of the qsd operon involved in AHLs degradation.

### 2.5. Rhodococcal Quorum-Quenching of Rhizobial AHL Communication Was Responsible for the Observed Changes in the Biofilm

#### 2.5.1. A *Rhodococcus* Mutant with A Deletion of the AHL-QsdA Lactonase Gene Lost Its Full Ability to Inhibit the Formation of Rhizobial Biofilms

**Figure 5:**
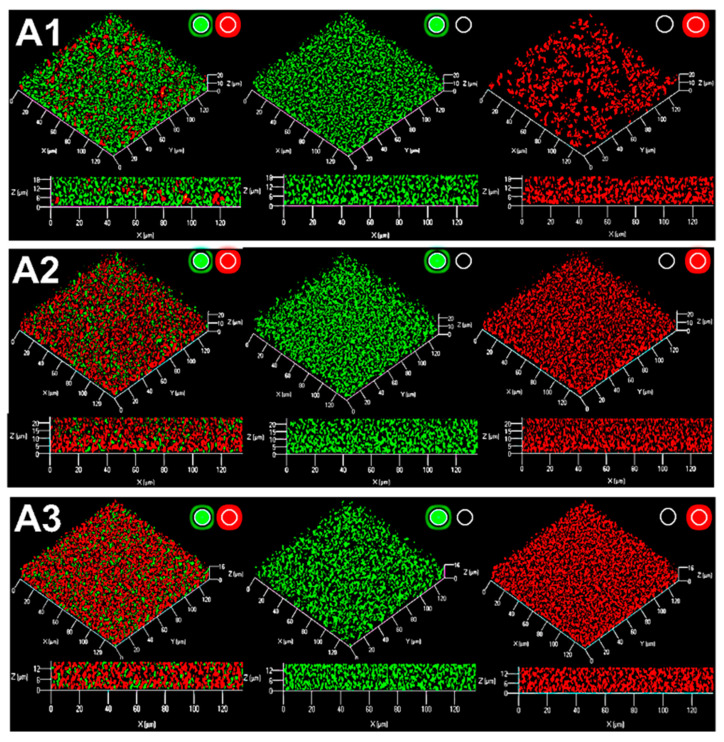

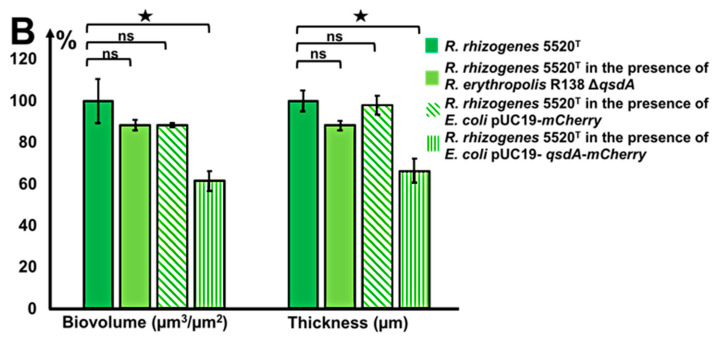
Effects of the *R. erythropolis* ΔqsdA deletion mutant and of QsdA AHL-lactonase-producing *E. coli* strains on *R. rhizogenes* biofilm formation. CLSM analysis of the dual-species biofilm formed by *R. rhizogenes* 5520^T^ (green fluorescence) and *R. erythropolis* R138 (or *E. coli*) (red fluorescence) (1:1 ratio). *R. rhizogenes* and rhodococcal (or *E. coli*) bacteria were tagged with GFP and mCherry via the pHC60-gfp and pEPR1(or pUC19)-mCherry vectors, respectively. 3D representation and side view of the dual-species biofilm formed by *R. rhizogenes* and *R. erythropolis* ΔqsdA (**A1**), *R. rhizogenes* and *E. coli* pUC19-mCherry (**A2**), and *R. rhizogenes* and *E. coli* pUC19-qsdA-mCherry (**A3**) (activatation of the green or red channel is indicated by a light spot of the corresponding color). COMSTAT analysis of the biovolume and thickness of the biofilms developed. *R. rhizogenes* biofilm, biovolume, and thickness values were set to 100%, for use as a reference in comparison with mixed biofilm conditions (**B**). The data shown are the means of three measurements from three independent experiments. Significant differences in biovolume and thickness values (Mann–Whitney test; *p*-value < 0.01) are indicated by asterisks (★ *p* < 0.05); ns, not significant.

The QsdA lactonase (E.C. 3.1.1.81) has been identified as the key enzyme of the *qsd* operon for the catabolism of diverse AHLs [53,60]. A *qsdA* deletion mutant was, therefore, constructed in a previous study investigating the role of the Qsd pathway in controlling tuber soft-rot due to *P. atrosepticum* [61]. The presence of a single copy of the *qsdA* gene in the genome of *R. erythropolis* R138 was checked by Southern blotting with a *qsdA* probe [61]. Here, the *R. erythropolis* R138 Δ*qsdA* strain was tagged with the mCherry fluorophore, resulting in red fluorescence labeling, while *R. rhizogenes* cells were green (Figure 5A1). Ability of the *R. erythropolis* R138 Δ*qsdA* strain to inhibit rhizobial biofilm formation was compared with that of the parental strain. The presence of the R138 Δ*qsdA* strain in the mixed biofilm led to non-significant modifications of *R. rhizogenes* biofilm biovolume and thickness (Figure 5).

#### 2.5.2. A Heterologous Host Carrying the Gene Encoding the Rhodococcal QsdA Lactonase Acquired the Ability to Inhibit Formation of the Rhizobial Biofilm

The role of the *qsdA* gene in QQ activity was previously assessed by introducing this gene into *Escherichia coli*, a bacterium unable to degrade AHLs. The *qsdA* gene is located on the high-copy number pUC19 plasmid (Table S1), and the heterologous expression of QsdA was checked [61]. The transformed strain strongly degrades AHLs and was found to have acquired the concomitant biocontrol activity against *P. atrosepticum* harbored by the biocontrol agent R138 [61]. The QsdA-expressing *E. coli* strain DH5α(pUC19-*qsdA*) and the control *E. coli* DH5α strain carrying the empty pUC19 vector were tagged with the mCherry fluorophore, and their ability to inhibit rhizobial biofilm formation was then compared.

Unlike rhodococci, the distribution of *E. coli* cells in the mixed biofilm is homogeneous (Figure 5A1 vs. Figure 5A2,A3). In the presence of *E. coli* DH5α(pUC19-*qsdA*), the mixed biofilm revealed a significant decrease in biovolume and thickness values of the *R. rhizogenes* strain. A 38% decrease in biovolume and a 33% decrease in thickness relative to the single-species *R. rhizogenes* biofilm were observed for the mixed *E. coli* DH5α(pUC19-qsdA)-*R. rhizogenes* biofilm (Figure 5B). The values decreased from 23.12 to 14.25 µm^3^/µm^2^ for biovolume and from 23.93 to 15.40 µm for thickness. By contrast, no significant decrease in biovolume or thickness was observed for the mixed *E. coli* DH5α(pUC19)-*R. rhizogenes* biofilm (Figure 5B), confirming that bacterial strains producing the QsdA AHL-lactonase have biofilm-inhibiting properties, including in a mixed biofilm with varied structures.

#### 2.5.3. The Activity of the Rhodococcal Lactonase QsdA Was Sufficient to Inhibit Formation of the Rhizobial Biofilm

**Figure 6:**
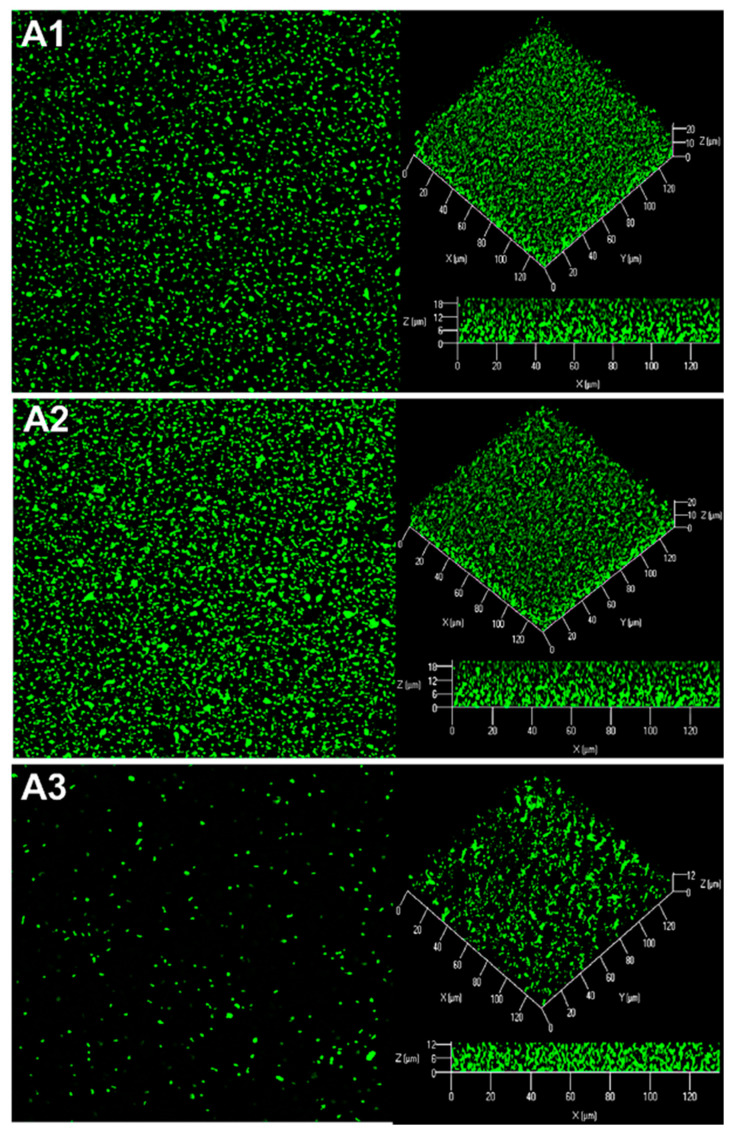

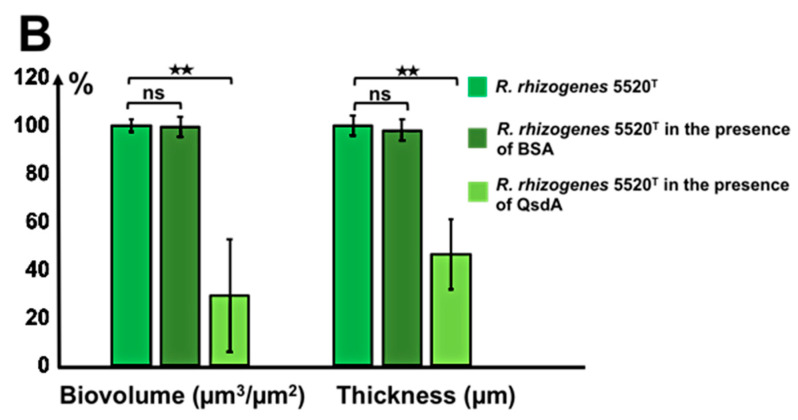
Effect of the purified AHL-lactonase QsdA on *R. rhizogenes* biofilm formation. Control conditions for biofilm formatin were established by substituting QsdA by bovine serum albumin (BSA). *R. rhizogenes* was transformed with the pHC60-gfp plasmid to tag bacteria by the constitutive expression of gfp (green fluorescent cells). Top view, 3D representation and side view of the *R. rhizogenes* biofilm in the presence of 100 µL of elution buffer alone (**A1**) or containing the BSA (**A2**) or the QsdA lactonase (**A3**). COMSTAT analysis of the biovolume and thickness of the biofilms developed (**B**). The data shown are the means of three measurements from three independent experiments. Significant differences in biovolume and thickness values (Mann–Whitney test; *p*-value < 0.01) are indicated by asterisks (★★ *p* < 0.01); ns, not significant.

The lactonase QsdA was overproduced and purified as described in the Materials and Methods section. The purified lactonase QsdA was added to a *R. rhizogenes* 5520^T^ culture to determine the effect of this QQ enzyme on *R. rhizogenes* biofilm structure. Control conditions without the enzyme were set up by replacing the QsdA enzyme with bovine serum albumin (BSA) protein. The addition of QsdA resulted in a decrease in biofilm biovolume from 24.22 ± 0.63 to 8.72 ± 2.17 µm^3^/µm^2^, revealing 64% biofilm inhibition (Figure 6). Biofilm thickness was also affected by the presence of QsdA, which led to a decrease in thickness from 23.42 ± 0.93 to 11.94 ± 1.07 µm. This finding confirms the impact of QQ as a mechanism inhibiting biofilm formation and reveals the potential role of this powerful AHL-lactonase in biofilm prevention.

## 3. Discussion

QS systems control diverse functions requiring the concerted actions of numerous individuals. They are cell-to-cell communication systems based on both the synthesis and perception of signaling molecules, the best known of which belong to the AHL family [17,18]. QS and biofilm formation are closely interconnected features of the social life of bacteria, particularly during the switch from a planktonic to a sessile lifestyle [8,11]. This synchronization appears to be essential at all stages of biofilm development, for metabolic reasons in particular. For example, swarming motility, which underlies surface motility within the developing biofilm at early stages of its development, has been shown to be under the control of QS regulation and nutrient conditions [62,63]. In addition, throughout biofilm construction, the secretion of a high density of extracellular polymeric substances (EPS) enables the cells to activate EPS synthesis selectively in the biofilm, thereby decreasing the costs of EPS production relative to the planktonic phase [64]. A downregulation of EPS synthesis at high cell density can also allow attached cells to redirect energy from EPS production to growth and cell division before a dispersal event [64]. Finally, QS triggers biofilm dispersion in a coordinated manner, probably because biofilm disassembly remains essential to allow bacteria to escape and colonize new niches in conditions of nutrient limitation [16].

AHLs are signaling molecules produced by various Gram-negative bacteria [17,18]. Among them, many phytopathogens use AHL-based communication [50,65], including a large number of rhizobial strains described as biofilm formers, in which QS plays a crucial role in the switch in lifestyle towards biofilms. This switch, governed by QS, involves modifications to the bacterial phenotype, including bacterial motility [66,67] and EPS production [68,69,70]. *R. rhizogenes* produces bulky biofilms responsible for economically significant damage to vegetables, such as tomatoes [42,44,48]. This bacterium was therefore considered a relevant model for this study. HPLC–MS characterization of the AHLs produced by *R. rhizogenes* 5520^T^ showed the production principally of 3-OH-C_14:1_-HSL and, to a lesser extent, 3-OH-C_14_-HSL (Figure 1). On the basis of a previous analysis, we suggest that 3-OH-C_14:1_-HSL is the only one of these molecules acting as a QS signal, and that 3-OH-C_14_-HSL is a less specific product of AHL synthase or a catabolite that appears during AHL turnover [58,71,72]. Interestingly, 3-OH-C_14:1_-HSL also appears to be the master AHL in the symbiotic nodule-forming *Rhizobium leguminosarum* species [49,51,52,73]. This apparent incongruity has been explained, in particular, by Velazquez et al. [74,75], who showed that the coexistence of symbiosis- and pathogenicity-determining genes in *R. rhizogenes* strains enables these bacteria to induce either beneficial (nodules) or deleterious (tumors or hairy roots) effects in plants.

AHL communication has been studied in *R. rhizogenes* in the context of biofilm establishment *in vitro*. Confocal microscopy and promoter-probe vectors carrying fluorescent protein-reporter genes are classical tools that have been used to monitor biofilm formation, structure, and development [76,77,78,79,80,81,82]. They are also powerful tools for localizing and quantifying the AHL-based communication of various plant-associated Gram-negative bacteria [57,83,84,85,86,87,88]. We used the fine detection capacity of the *P. atrosepticum* 6276-EI AHL biosensor to show that *R. rhizogenes* strain 5520^T^ produces AHLs in the biofilm context (Figure 3). This pectobacterial biosensor colonized the rhizobial biofilm only weakly, but its presence was uniform within biofilm, and the green fluorescence associated with AHL production was detected both in the center and periphery of the biofilm, demonstrating the diffusion of these signaling molecules through the exopolysaccharide matrix (Figure 3).

In the plant rhizosphere, the triggering of disease by QS is disrupted by the presence of both bacteria producing QS signals and quencher bacteria capable of interfering with signal exchanges [89]. Diverse bacteria from the α-, β- and γ-Proteobacteria; Firmicutes; and Actinobacteria have been shown to inactivate QS signals [27,90,91,92]. Actinobacteria from the genus *Rhodococcus* have been shown to degrade various QS signals, including AHLs [89,91,93]. In *R. erythropolis* strain R138, recent advances in our understanding of the mechanisms regulating expression of the *qsd* operon expression made it possible to remove a fundamental technological lock and to construct the first biosensor of AHL-based QQ activity [53]. A dual-color rhodococcal reporter, encoding the mCherry fluorophore as a cell tag and GFP as a regulator-based reporter system, has been used for the simultaneous visualization in planta of bacterial spread and of the QQ activity, leading to the biocontrol of potato soft rot [59,94]. This work revealed the chronology of events leading to tuber maceration and QS communication, in addition to the disruption of QS communication and concomitant protection of the plant by the rhodococcal biocontrol agent. Here, we used the same biosensor to sense both QS and QQ activities within the rhizobial biofilm (Figure 4). Rhodococcal cells may adopt two different shapes, rods and cocci, depending on their metabolic state. The rod-shaped cells reflect an active metabolic state, whereas the coccal cells correspond to an inactive metabolic state [95,96]. In our study, most of the yellow rhodococcal cells (quenchers) were rod-shaped, whereas most of the red rhodococcal cells were coccoid, this difference potentially reflecting the catabolism of AHLs in the quencher cells. Finally, such AHL catabolism was observed, particularly in regions containing a mixture of large numbers of rhizobial and rhodococcal cells. The proximity of partners is probably required for the entry of the AHL into the rhodococci destined for degradation. These are, to our knowledge, the first images of QQ activity within a mixed biofilm.

The presence of the R138 biocontrol agent led to changes in the characteristics of the biofilm formed by the *R. rhizogenes* phytopathogen (Figure 2). These modifications included changes in both the biovolume and structure of the biofilm. They are thought to be related to disruption of the QS communication between rhizobia in the biofilm. One possible interpretation of this phenomenon is that the jamming of AHL communication has a severe effect on the metabolism of rhizobial EPS, as previously observed in the *Pseudomonas aeruginosa* model [64]. This assertion is also based on the demonstration that EPS metabolism is essential for cell attachment and normal biofilm formation in another strain of *R. rhizogenes* [97].

The role of the Qsd pathway in modifying biofilm traits was demonstrated by studying the activity of the AHL-lactonase QsdA. The functions of QsdA, the key enzyme in this catabolic pathway, were previously investigated by transferring the *qsdA* gene to a heterologous host (*E. coli*), which transformed this bacterium into a powerful AHL quencher [61]. We also constructed an *R. erythropolis* R138 *qsdA* deletion mutant with an impaired ability to break down AHL [61]. As expected, the mutated strain completely lost its ability to alter biofilm formation by *R. rhizogenes*; conversely, the transformed *E. coli* strain acquired this ability following insertion of the R138 *qsdA* gene (Figure 5). Interestingly, deletion of the *qsdA* gene did not completely abolish the biofilm-inhibiting effect of *R. erythropolis* R138. This probably reflects the activity, in strain R138, of other enzymes involved in AHL catabolism, such as an AHL-amidohydrolase and at least one type of AHL lactonase from the α/β hydrolase family [36,53,98,99,100,101]. Nevertheless, QsdA lactonase, even following isolation from its progenitor, had strong efficacy against biofilm formation, similar to that recorded with the R138 strain or the heterologous host *E. coli*, under our assay conditions. These findings confirm the significant potential for QQ already observed for other AHL lactonases of the phosphotriesterase family, to which QsdA belongs [24,102,103].

In conclusion, this work shows that biofilm formation by *R. rhizogenes* is partly QS-dependent and that a biocontrol strategy based on another organism with QQ ability could be used to target this species. Future studies are nevertheless required to determine the physicochemical characteristics of QsdA (e.g., thermal stability, catalytic temperature range, resistance to detergents and organic solvents, radiation and proteases), to evaluate its true potential, in freeze-dried formulations for example [41]. Finally, additional support for the use of strain R138 as a biocontrol agent [54,55,99] is provided by the demonstration of its ability to prevent biofilm formation and biofouling in this study.

## 4. Materials and Methods

### 4.1. Bacterial Strains and Culture Conditions

The bacterial strains and plasmids used in this study are listed in Table S1. All strains were routinely grown in Luria–Bertani (LB) medium, on a rotary shaker (180 rpm) at 25 °C, except for *E. coli* transformants and the AHL biosensor strain *E. coli* pSB401, which were cultured at 37 and 30 °C, respectively. Growth was monitored by spectrophotometry at 580 nm. All cultures were inoculated at an initial OD_580_ of 0.05. The *R. rhizogenes*-type strain 5520^T^ strain was labeled with GFP by introducing the pHC60-*gfp* plasmid by electrotransformation, as described by Mersereau et al. [104]. The introduction of pHC60-*gfp* led to the constitutive expression of *gfp* by rhizobial cells. The biocontrol agent *R. erythropolis* R138 and its Δ*qsdA* isogenic mutant were constitutively labeled with mCherry fluorescence by introducing the pEPR1–*mCherry* vector, by electric field-mediated transformation, as described by Barbey et al. [61]. The previously described *R. erythropolis* pEPR1-*qsdR*-P*qsd::gfp*-*mCherry* strain was used to monitor the QQ activity of this phytoprotective agent [53]. AHL production was detected with *E. coli* pSB401 or *P. atrosepticum* 6276 pME6000-P*_luxI_*::*gfp*-*cfp*, as described by Chane et al. [38]. When necessary, antibiotics were added to the medium: tetracycline (Tc) at a concentration of 15 µg.mL^‒1^ for *R. rhizogenes* and *E. coli*, ampicillin (Ap, Sigma Aldrich, Saint Quentin Fallavier, France) at 100 µg.mL^‒1^ for *E. coli* and 200 µg.mL^−1^ for *R. erythropolis*, or kanamycin (Km) at 50 µg.mL^−1^ for *E. coli*.

### 4.2. Extraction and Characterization of the AHL Signaling Molecules Produced by R. rhizogenes 5520^T^

AHLs were extracted from the supernatants of *R. rhizogenes* 5520^T^ stationary phase cultures in LB. Briefly, cells were removed by centrifugation, and AHLs were obtained by two successive extractions from 1 mL of supernatant with ethyl acetate 1:1 (v:v). Ethyl acetate was removed by rotary evaporation and the extracts were reconstituted in 500 µL acetonitrile for LCMS analyses. Extracts were loaded onto a Gemini C6 phenyl column (250 × 4.6 mm, 5 µm) from Phenomenex installed on an Ultimate 3000 chromatographic system (Dionex, ThermoFisher Scientific, Illkirch-Graffenstaden, France). Separations were performed at a flow rate of 0.6 mL/min at 40 °C. The solvents for elution were water/acetonitrile 95:5 (A) and water/acetonitrile 2:98 (B), both supplemented with 10 mM ammonium acetate. The elution program was as follows: 30 to 100% B over 15 min, and then 100% B for 5 min.

Detection was performed with a micrOTOF-QII quadrupole time-of-flight mass spectrometer (QqTOF-MS, Bruker Daltonics, Wissembourg, France) from Bruker Daltonics. This instrument is equipped with an electrospray ionization source. The mass spectrometer was calibrated with a mixture of 10 mM NaOH and 0.2% (v:v) formic acid in water in order to achieve a mass accuracy of 5 ppm. Analyses were performed in positive mode with an *m*/*z* range of 50–1000. The capillary voltage of the ion source was set to 4500 V, the nebulizer gas pressure was 2.8 bars, and the dry gas flow rate was 12 L.min^−1^. The dry temperature was set at 200 °C. Compass HyStar 3.2 (Bruker Daltonics, Wissembourg, France) was used to couple chromatography to spectrometry, otofControl 4.0 (Bruker Daltonics, Wissembourg, France) was used for QqTOF-MS operation, and DataAnalysis 4.3 (Bruker Daltonics, Wissembourg, France) was used for data processing, all from Bruker Daltonics.

Chromatographic retention time and *m*/*z* at high mass resolution were compared with those of synthetic AHL standards: 3-oxo-C_8_-HSL, 3 OH-C_8_-HSL, 3-OH-C_14:1_-HSL (BertinPharma, Montigny le Bretonneux, France), 3-oxo-C_14_-HSL, and 3-OH-C_14_-HSL (Sigma Aldrich, Saint Quentin Fallavier, France). The concentrations of each AHL were determined by comparison with standards at various concentrations ranging from 0.05 to 10 mg.L^−1^.

### 4.3. Construction of mCherry-Tagged E. coli Strains

The *mCherry* cassette was inserted, as a 1011-bp *Pst*I-*Hin*dIII fragment, into the pUC19 vector and the pUC19-*qsdA* vector*,* constructed as previously described by Barbey et al. [61] (Table S1). PCR amplification was performed with the Q5 DNA polymerase (New England Biolabs, Evry, France) and the following primers: 5′TAATAAAAGCTTCCATTATGACTGGTTGGGCT3′ and 5′TAATAACTGCAGGAATTCTTATTTGTAGAGTTCATCCA3′. After enzymatic digestion with *Pst*I (New England Biolabs, Evry, France) and *Hin*dIII (New England Biolabs, Evry, France), the vectors and PCR products were ligated with the T4 DNA ligase (New England Biolabs, Evry, France), according to the manufacturer’s instructions. The *E. coli* Top10 strain was then transformed with the ligation products, by the standard heat-shock method, at 42 °C for 45 s. After 1.5 h of incubation in LB at 37 °C, the transformed cells were plated on selective medium supplemented with the appropriate antibiotic and incubated for 24 h at 37 °C. The transformant nature of the colonies obtained was verified by PCR with plasmid-specific primers flanking the *mCherry* gene insertion site. Finally, the recombinant pUC19–*qsdA*–*mCherry* and pUC19–*mCherry* plasmids were sequenced to check for the absence of mutations in the *mCherry* cassette.

### 4.4. Extraction and Purification of the QsdA Lactonase Enzyme

The *E. coli* BL21 (DE3) strain containing the pET22-*qsdA* plasmid was obtained as described by Barbey et al. [105] (Table S1). QsdA was produced as described by Barbey et al. [53]. The purified His-tagged QsdA protein was then checked by one-dimensional denaturing sodium dodecyl sulfate polyacrylamide gel electrophoresis (SDS-PAGE) in a 12% acrylamide (29:1 acrylamide/bisacrylamide, Eurobio) resolving gel with a 7% acrylamide stacking gel. We used Bio-Rad Precision plus protein TM standard markers (Bio-Rad, Marnes La Coquette, France) to determine the molecular weight of the protein. After electrophoresis in a mini-PROTEAN^®^ 3 cell (Bio-Rad, Marnes La Coquette, France), the gel was stained with Coomassie Brilliant Blue R250 (Sigma Aldrich, Saint Quentin Fallavier, France) for protein visualization. A single protein band with a molecular mass of about 35 kDa was excised from the gel and subjected to in-gel trypsin digestion, as previously described [105]. The AHL activity of QsdA was assessed with the AHL biosensor *E. coli* pSB401. We incubated 24 µg (i.e., 6.74 × 10^−4^ µmol) QsdA in TYP medium (2 g.L^−1^ yeast extract, 5 g.L^−1^ tryptone, 0.2 g.L^−1^ NaH_2_PO_4_, and 0.3 g.L^−1^ KH_2_PO_4_) supplemented with 25 µM 3-OH-C_14:1_-HSL for 24 h at 25 °C. As a negative control for AHL degradation, TYP medium containing 25 µM 3-OH-C_14:1_-HSL was also incubated without QsdA for 24 h at 25 °C. After incubation, AHL determinations were performed by mixing a volume of 10 µL of each testing solution with a culture of 100 µL *E. coli* pSB401 at an OD_580nm_ of 0.1 in the wells of a white 96-well microplate and incubating at 30 °C for 24 h (Thermo Scientific Nunc 96-Well Flat-Bottom Plate, ThermoFisher Scientific, Illkirch-Graffenstaden, France), with measurements of bioluminescence and absorbance at 580 nm every hour. The presence of 3-OH-C_14:1_-HSL induced transcription from the P_LuxI_ promoter, leading to bioluminescence due to *luxCDABE* expression. An absence of bioluminescence was taken to indicate that the 3-OH-C_14:1_-HSL had been degraded.

### 4.5. Biofilm Culture

The ability of *R. erythropolis* R138 to prevent *R. rhizogenes* biofilm formation was studied by growing single- or dual-species biofilms under static conditions, in 24-well microplates with glass bottoms (Sensoplate, Greiner Bio-One, Les Ulis, France). Each partner strain (*R. rhizogenes* 5520^T^, *R. erythropolis* R138, or *P. atrosepticum* 6276-EI) was grown separately overnight in LB medium. The bacterial cultures were washed twice to eliminate antibiotics, resuspended in LB medium, and mixed such that the OD_580nm_ of each strain was 0.1 (about 1 × 10^8^ CFU.mL^−1^). The plates were then incubated at 25 °C for 48 h and rinsed twice with saline for CLSM observation. The strain lacking a fluorescent plasmid tag was stained with 5 µM SYTO 61 dye (Invitrogen, ThermoFisher, Illkirch-Graffenstaden, France). The effect of QsdA on *R. rhizogenes* biofilm structure was studied by adding 24 µg (i.e., 6.74 × 10^−4^ µmol or concentration of 24 µg.mL^−1^). The control condition was performed by replacing QsdA by the same number of moles of BSA.

### 4.6. Confocal Laser Scanning Microscopy (CLSM)

Biofilm observations were performed with a Zeiss LSM710 confocal laser scanning microscope (Zeiss, Germany) equipped with a × 63 oil immersion objective. Bacteria were detected by monitoring GFP fluorescence or mCherry fluorescence with the following excitation and emission wavelengths: GFP (excitation at 488 nm and emission at 509 nm; green channel) and mCherry (excitation at 543 nm and emission at 610 nm; red channel). The fluorescence signals of double-labeled specimens were acquired simultaneously. Images were taken at 1 µm intervals throughout the entire depth of the biofilm. Three-dimensional (3D) imaging data were visualized and processed with Zen 2.1 software (Zeiss, Germany), using the same gain and offset parameters for all images. Quantitative analyses of image stacks were performed with ImageJ (https://imagej.nih.gov/ij/; 15 November 2020) and COMSTAT software (http://www.imageanalysis.dk/; 15 November 2020) [56]. Experiments were performed at least three times, with three independent replicates for each experiment.

### 4.7. Antibiosis Test

Antibiosis experiments were performed by completely covering solid LB with a stationary phase culture of *R. rhizogenes* or *R. erythropolis*. The medium was allowed to dry, and a volume of 10 µL of each culture was then dispensed onto its surface. Non-inoculated LB was dispensed onto the surface of the medium for the negative control. Agar plates were incubated at 25 °C for 24 h.

### 4.8. Statistical Analysis

Past software (PAleontological STatistics) was used for statistical analyses. Two-tailed unpaired Mann–Whitney tests were performed, and *p*-values were calculated. Means and SEM values were calculated and plotted.

## Data Availability

Not applicable.

## Supplementary Materials

The following are available online at https://www.mdpi.com/article/10.3390/ijms22158241/s1.
