# Supplementary material for: Biocontrol of Biofilm Formation: Jamming of Sessile-Associated Rhizobial Communication by Rhodococcal Quorum-Quenching
Source: Int J Mol Sci. 2021 Jul 31;22(15):8241. doi: 10.3390/ijms22158241 (PMC8347015; doi:10.3390/ijms22158241)
Supplement: Supplementary file 1 [file ijms-22-08241-s001.zip › Bourigaultetal-IJMS 2021-Figure S2..pdf]

International Journal of *Molecular Sciences*

Article

Biocontrol of Biofilm Formation: Jamming Sessile-Associated Rhizobial Communication by Rhodococcal Quorum-Quenching

Yvann Bourigault [1,2], Sophie Rodrigues [3], Alexandre Crépin [4], Andrea Chane [1], Laure Taupin [3], Mathilde Bouteiller [1,2], Charly Dupont [1,2], Annabelle Merieau [1,2], Yoan Konto-Ghiorghi [1,2], Amine M. Boukerb [1], Marie Turner [5,6], Céline Hamon [5], Alain Dufour [3], Corinne Barbey [1,2], and Xavier Latour [1,2,6*]

1 Laboratory of Microbiology Signals and Microenvironment (LMSM EA 4312), University of Rouen Normandy, 55 rue Saint-Germain, F-27000 Evreux, France; yvann.bourigault@univ-rouen.fr (Y.B.); corinne.barbey@univ-rouen.fr (C.B.); chane.andrea@gmail.com (A.C.); mathilde.bouteiller7@univ-rouen.fr (M.B.); charly.dupont7@univ-rouen.fr (C.D.); annabelle.merieau@univ-rouen.fr (A.M.); yoan.konto-ghiorghi@univ-rouen.fr (Y.K-G); amine.boukerb@univ-rouen.fr (A.M.B.)
2 Research Federations NORVEGE Fed4277 & NORSEVE, Normandy University, F-76821 Mont-Saint-Aignan, France
3 Université de Bretagne-Sud, EA 3884, LBCM, IUEM, F-56100 Lorient, France; sophie.rodrigues@univ-ubs.fr (S.R.); laure.taupin@univ-ubs.fr (L.T) ; alain.dufour@univ-ubs.fr (A.D.)
4 Laboratoire Ecologie et Biologie des Interactions, UMR CNRS 7267 F-86073 Poitiers, France; alexandre.crepin@univ-poitiers.fr (A.CR)
5 Végénov, F-29250 Saint-Pol-de-Léon, France; turner@vegenov.com (M.T.); hamon@vegenov.com (C.H.)
6 Biocontrol Consortium, F-75007 Paris, France
* Correspondence: xavier.latour@univ-rouen.fr; +33-235-146-000 (X.L.)

Supplementary Material

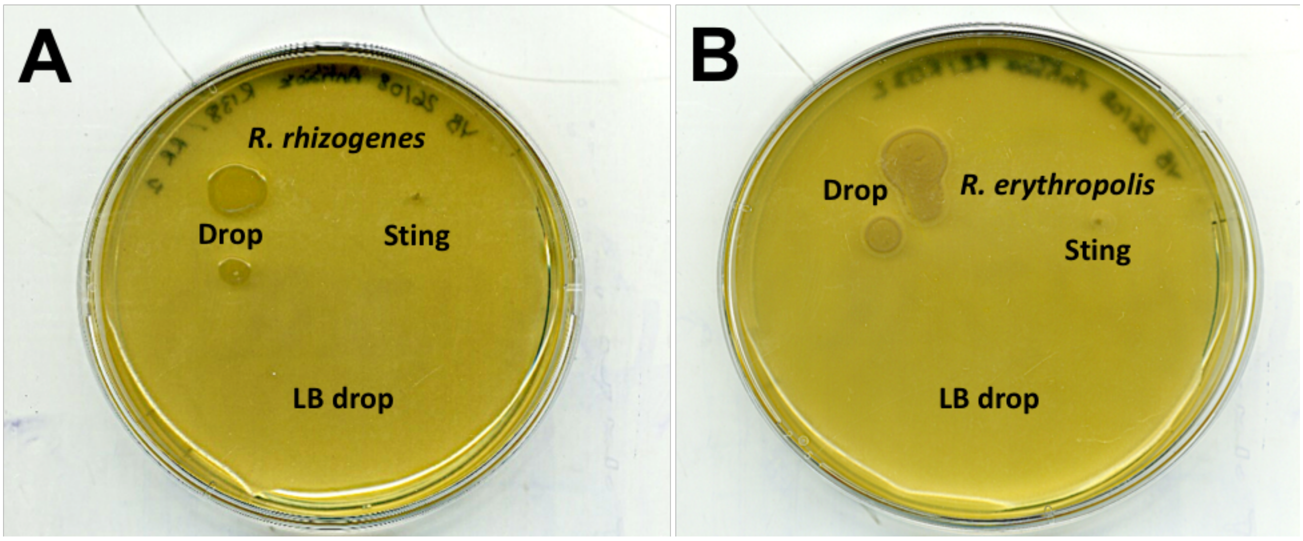

Figure S2. Screening for direct antagonism between *R. rhizogenes* 5520^T and *R. erythropolis* R138. Antibiosis tests were performed by completely covering LB medium with a stationary phase culture of *R. rhizogenes* culture (**A**), or *R. erythropolis* (**B**). Then, a volume of 10 µl of each culture was dropped on the dried medium. The non-inoculated LB broth used to perform bacterial cultures was dropped as a negative control, (see also Materials and Methods Section). Antibiosis tests were repeated at least three times.
