# Supplementary material for: Biocontrol of Biofilm Formation: Jamming of Sessile-Associated Rhizobial Communication by Rhodococcal Quorum-Quenching
Source: Int J Mol Sci. 2021 Jul 31;22(15):8241. doi: 10.3390/ijms22158241 (PMC8347015; doi:10.3390/ijms22158241)
Supplement: Supplementary file 1 [file ijms-22-08241-s001.zip › Bourigaultetal-IJMS 2021-Figure S6..pdf]

International Journal of *Molecular Sciences*

MDPI

Article

Biocontrol of Biofilm Formation: Jamming Sessile-Associated Rhizobial Communication by Rhodococcal Quorum-Quenching

Yvann Bourigault [1,2], Sophie Rodrigues [3], Alexandre Crépin [4], Andrea Chane [1], Laure Taupin [3], Mathilde Bouteiller [1,2], Charly Dupont [1,2], Annabelle Merieau [1,2], Yoan Konto-Ghiorghi [1,2], Amine M. Boukerb [1], Marie Turner [5,6], Céline Hamon [5], Alain Dufour [3], Corinne Barbey [1,2], and Xavier Latour [1,2,6*]

1 Laboratory of Microbiology Signals and Microenvironment (LMSM EA 4312), University of Rouen Normandy, 55 rue Saint-Germain, F-27000 Evreux, France; yvann.bourigault@univ-rouen.fr (Y.B.); corinne.barbey@univ-rouen.fr (C.B.); chane.andrea@gmail.com (A.C.); mathilde.bouteiller7@univ-rouen.fr (M.B.); charly.dupont7@univ-rouen.fr (C.D.); annabelle.merieau@univ-rouen.fr (A.M.); yoan.konto-ghiorghi@univ-rouen.fr (Y.K-G); amine.boukerb@univ-rouen.fr (A.M.B.)

2 Research Federations NORVEGE Fed4277 & NORSEVE, Normandy University, F-76821 Mont-Saint-Aignan, France

3 Université de Bretagne-Sud, EA 3884, LBCM, IUEM, F-56100 Lorient, France; sophie.rodrigues@univ-ubs.fr (S.R.); laure.taupin@univ-ubs.fr (L.T) ; alain.dufour@univ-ubs.fr (A.D.)

4 Laboratoire Ecologie et Biologie des Interactions, UMR CNRS 7267 F-86073 Poitiers, France; alexandre.crepin@univ-poitiers.fr (A.CR)

5 Végénov, F-29250 Saint-Pol-de-Léon, France; turner@vegenov.com (M.T.); hamon@vegenov.com (C.H.)

6 Biocontrol Consortium, F-75007 Paris, France

* Correspondence: xavier.latour@univ-rouen.fr; +33-235-146-000 (X.L.)

Supplementary Material

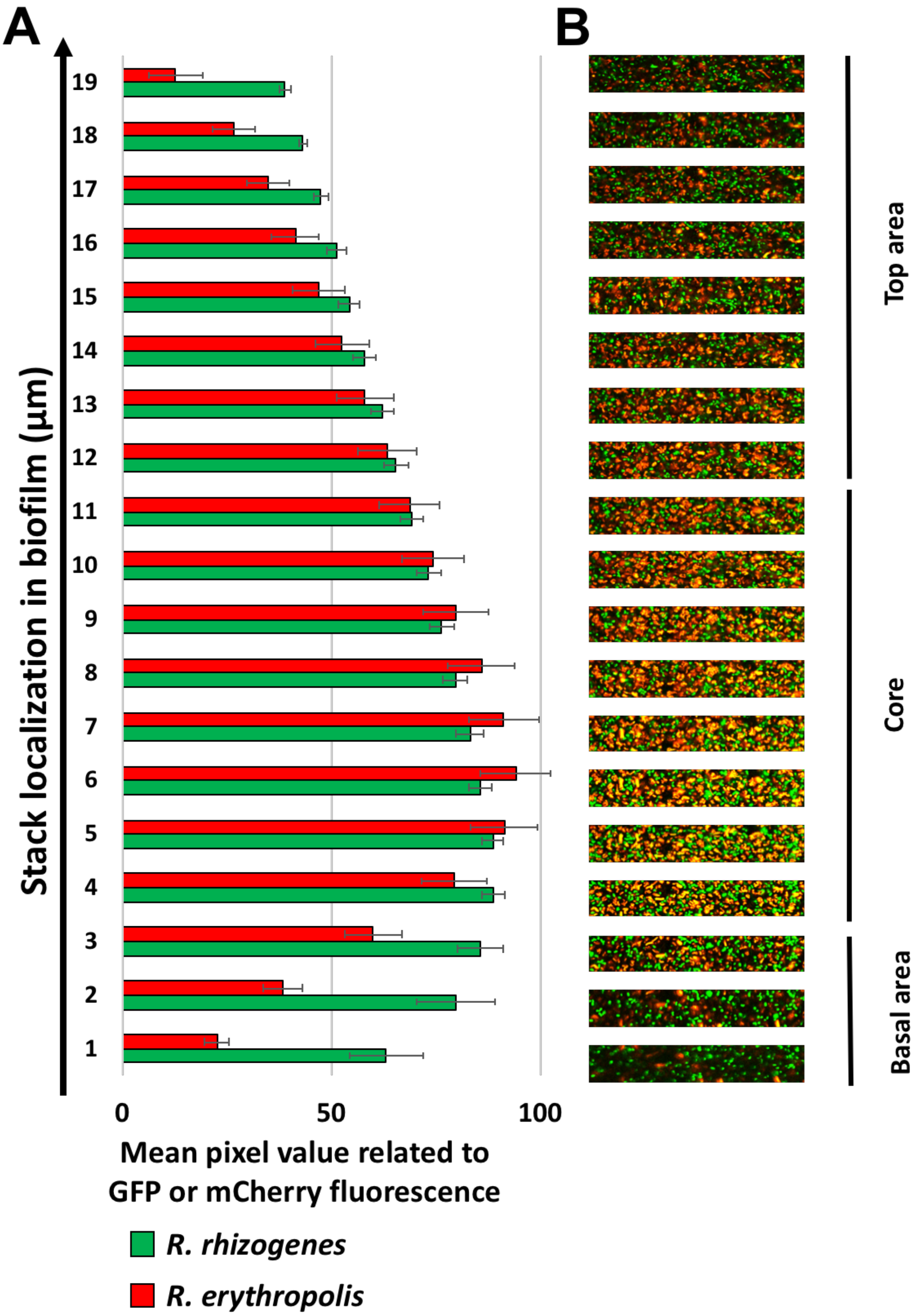

Figure S6. Distribution of *R. rhizogenes* 5520ᵀ and *R. erythropolis* R138 cell patterns within the biofilm. Confocal laser scanning microscopy (CLSM) analysis of the dual species biofilm formed by *R. rhizogenes* 5520ᵀ and *R. erythropolis* R138 was achieved at an inoculation ratio of 1:1. *R. rhizogenes* 5520ᵀ and *R. erythropolis* R138 bacteria were tagged with GFP and mCherry *via* the pHC60-*gfp* and pEPR1-*qsdR*-P*qsd*::*gfp*-*mCherry* vectors, respectively. (**A**) Pixel quantification of each of the nineteen stacks representing the entire biofilm structure. Each bar represents the pixel quantification value related to the GFP or mCherry signal detected using the 2D CLSM images of the dual species biofilm. (**B**) Related 2D CLSM images of each pixel quantification value for the dual species biofilm. The data shown are the means of at least three measurements from three independent experiments.
