# Supplementary material for: Biocontrol of Biofilm Formation: Jamming of Sessile-Associated Rhizobial Communication by Rhodococcal Quorum-Quenching
Source: Int J Mol Sci. 2021 Jul 31;22(15):8241. doi: 10.3390/ijms22158241 (PMC8347015; doi:10.3390/ijms22158241)
Supplement: Supplementary file 1 [file ijms-22-08241-s001.zip › Bourigaultetal-IJMS 2021-Table S1..pdf]

International Journal of
Molecular Sciences

MDPI

Article

Biocontrol of Biofilm Formation: Jamming Sessile-Associated Rhizobial Communication by Rhodococcal Quorum-Quenching

Yvann Bourigault [1,2], Sophie Rodrigues [3], Alexandre Crépin [4], Andrea Chane [1], Laure Taupin [3], Mathilde Bouteiller [1,2], Charly Dupont [1,2], Annabelle Merieau [1,2], Yoan Konto-Ghiorghi [1,2], Amine M. Boukerb [1], Marie Turner [5,6], Céline Hamon [5], Alain Dufour [3], Corinne Barbey [1,2], and Xavier Latour [1,2,6*]

[1] Laboratory of Microbiology Signals and Microenvironment (LMSM EA 4312), University of Rouen Normandy, 55 rue Saint-Germain, F-27000 Evreux, France; yvann.bourigault@univ-rouen.fr (Y.B.); corinne.barbey@univ-rouen.fr (C.B.); chane.andrea@gmail.com (A.C.); mathilde.bouteiller7@univ-rouen.fr (M.B.); charly.dupont7@univ-rouen.fr (C.D.); annabelle.merieau@univ-rouen.fr (A.M.); yoan.konto-ghiorghi@univ-rouen.fr (Y.K-G); amine.boukerb@univ-rouen.fr (A.M.B.)
[2] Research Federations NORVEGE Fed4277 & NORSEVE, Normandy University, F-76821 Mont-Saint-Aignan, France
[3] Université de Bretagne-Sud, EA 3884, LBCM, IUEM, F-56100 Lorient, France; sophie.rodrigues@univ-ubs.fr (S.R.); laure.taupin@univ-ubs.fr (L.T) ; alain.dufour@univ-ubs.fr (A.D.)
[4] Laboratoire Ecologie et Biologie des Interactions, UMR CNRS 7267 F-86073 Poitiers, France; alexandre.crepin@univ-poitiers.fr (A.CR)
[5] Végénov, F-29250 Saint-Pol-de-Léon, France; turner@vegenov.com (M.T.); hamon@vegenov.com (C.H.)
[6] Biocontrol Consortium, F-75007 Paris, France
* Correspondence: xavier.latour@univ-rouen.fr; +33-235-146-000 (X.L.)

Supplementary Material

TABLE S1. Bacterial strains and plasmids used in this study

Strain or plasmid	Relevant characteristic(s)	Source or reference
Rhizobium rhizogenes		
5520^T	Type strain of *Rhizobium rhizogenes* species	Strain CFBP
5520^T(pHC60-*gfp*)	5520^T transformed by the pHC60 vector containing the *gfp* gene to label bacteria in green fluorescence; Tet^r	This study
Rhodococcus erythropolis		
R138	AHL-degrading isolate obtained from hydroponic culture of potato plants	[54]
R138 Δ*qsdA*	R138 with a 813 bp fragment deleted from the *qsdA* gene	[61]
R138 Δ*qsdA* (pEPR1-*mcherry)*	R138 Δ*qsdA* transformed by the pEPR1 *mcherry* plasmid to tag bacteria in red fluorescence; Km^r	[53]
R138 (pEPR1-*qsdR*-P*qsd::gfp*$_{uv}$*-mcherry)*	R138 strain transformed by pEPR1 *qsdR*-P*qsd::gfp*$_{uv}$*-mcherry* containing the transcriptional	[53]

	fusion P*qsd*::*gfp*$_{uv}$ to monitor the quorum quenching activity ; Km^{r}	
Pectobacterium atrosepticum		
6276	Potato soft rot pathogen, AHL producer	Strain CFBP, [106]
6276-EI	*luxI (syn. expI)* mutant derivative of CFBP 6276 strain unable to produce AHLs; Gm^{r}	[58]
6276-EI (pME6000-*luxR*-P$_{luxI}$*::gfp-cfp)*	*luxI (syn. expI)* mutant derivative of CFBP 6276 strain transformed by pME6000-*luxR*- P$_{luxI}$*::gfp-cfp* to monitor the quorum sensing activity; Tc^{r}	[38]
Escherichia coli		
DH5*α*	Host for cloning; SupE44 ΔlacU169 (Φ80lacZΔM15) hsdR17 recA1 endA1 gyrA96 thi-1 relA1	Lab. collection
DH5*α*(pUC19)	Strain DH5*α* carrying pUC19; Ap^{R}	[61]
DH5*α*(pUC19-*qsdA*)	QsdA-expressing DH5*α*; Ap^{R}	[61]
DH5*α*(pUC19-*qsdA*-*mCherry*	QsdA and mCherry expressing DH5*α*; Ap^{R}	This study
BL21 (DE3)	Strain transformed by the overexpression plasmid pET22-*qsdA* containing the lactonase encoding gene *qsdA*	[105]
**Plasmids**		
pHC60-*gfp*	Vector constitutively expressing the GFP	[104]
pME6000	Cloning vector, derivative of pVS1, low copy; Tc^{r}	[107]
pME6000 *luxR*-P$_{luxI}$*::gfp*$_{asv}$-*cfp*	pME6000-*cfp* with a P$_{luxI}$*::gfp*$_{asv}$ transcriptional fusion under the control of *luxR* expression; Tc^{r}	This study
pEPR1	Shuttle promoter-probe vector carrying the promoterless *gfp*$_{uv}$ reporter gene; Km^{r}	[108]
pEPR1-*mcherry*	pEPR1 vector containing a *mCherry* cassette under constitutive promoter; Km^{r}	This study
pEPR1-*qsdR*-P*qsd::gfp*$_{uv}$-*mcherry*	pEPR1-*mcherry* with a P*qsd::gfp*$_{uv}$ transcriptional fusion under the control of QsdR; Km^{r}	[53]
pET22-*qsdA*	Overexpression vector containing the *qsdA* gene; Ap^{R}	[105]
pUC19	Cloning vector for *E. coli*; Ap^{R}	Lab. collection
pUC19-*qsdA*	pUC19 containing the *qsdA* gene; Ap^{R}	[61]
pUC19-*mCherry*	Plasmid containing a mcherry cassette under constitutive promoter; Ap^{R}	This study

pUC19-*qsdA-mCherry*	Plasmid containing the *qsdA* gene and a mcherry cassette under constitutive promoter; Ap^R	This study

Km^r, Ap^r, Gm^r and Tc^r indicate resistance to kanamycin, ampicillin, gentamycin and tetracycline, respectively. AHL, *N*-acyl homoserine lactone. CFBP, Collection Française de Bactéries associées aux Plantes, Institut National de Recherche pour l'Agriculture, l'alimentation et l'Environnement (INRAE), Angers, France. FERA: The Food and Environment Research Agency, York, U.K.
